# Replacing Manual Planning of Whole Breast Irradiation With Knowledge-Based Automatic Optimization by Virtual Tangential-Fields Arc Therapy

**DOI:** 10.3389/fonc.2021.712423

**Published:** 2021-08-24

**Authors:** Roberta Castriconi, Pier Giorgio Esposito, Alessia Tudda, Paola Mangili, Sara Broggi, Andrei Fodor, Chiara L. Deantoni, Barbara Longobardi, Marcella Pasetti, Lucia Perna, Antonella del Vecchio, Nadia Gisella Di Muzio, Claudio Fiorino

**Affiliations:** ^1^Medical Physics, San Raffaele Scientific Institute, Milano, Italy; ^2^Radiotherapy, San Raffaele Scientific Institute, Milano, Italy

**Keywords:** breast cancer, radiation oncology, automation, plan optimization, tangential field, knowledge-based

## Abstract

**Purpose:**

To implement Knowledge Based (KB) automatic planning for right and left-sided whole breast treatment through a new volumetric technique (ViTAT, Virtual Tangential-fields Arc Therapy) mimicking conventional tangential fields (TF) irradiation.

**Materials and Method:**

A total of 193 clinical plans delivering TF with wedged or field-in-field beams were selected to train two KB-models for right(R) and left(L) sided breast cancer patients using the RapidPlan (RP) tool implemented in the Varian Eclipse system. Then, a template for ViTAT optimization, incorporating individual KB-optimized constraints, was interactively fine-tuned. ViTAT plans consisted of four arcs (6 MV) with start/stop angles consistent with the TF geometry variability within our population; the delivery was completely blocked along the arcs, apart from the first and last 20° of rotation for each arc. Optimized fine-tuned KB templates for automatic plan optimization were generated. Validation tests were performed on 60 new patients equally divided in R and L breast treatment: KB automatic ViTAT-plans (KB-ViTAT) were compared against the original TF plans in terms of OARs/PTVs dose-volume parameters. Wilcoxon-tests were used to assess the statistically significant differences.

**Results:**

KB models were successfully generated for both L and R sides. Overall, 1(3%) and 7(23%) out of 30 automatic KB-ViTAT plans were unacceptable compared to TF for R and L side, respectively. After the manual refinement of the start/stop angles, KB-ViTAT plans well fitted TF-performances for these patients as well. PTV coverage was comparable, while PTV D_1%_ was improved with KB-ViTAT by R:0.4/L:0.2 Gy (p < 0.05); ipsilateral OARs D_mean_ were similar with a slight (i.e., few % volume) improvement/worsening in the 15–35 Gy/2–15 Gy range, respectively. KB-ViTAT better spared contralateral OARs: D_mean_ of contralateral OARs was 0.1 Gy lower (p < 0.05); integral dose was R:5%/L:8% lower (p < 0.05) than TF. The overall time for the automatic plan optimization and final dose calculation was 12 ± 2 minutes.

**Conclusions:**

Fully automatic KB-optimization of ViTAT can efficiently replace manually optimized TF planning for whole breast irradiation. This approach was clinically implemented in our institute and may be suggested as a large-scale strategy for efficiently replacing manual planning with large sparing of time, elimination of inter-planner variability and of, seldomly occurring, sub-optimal manual plans.

## Introduction

Post-operative irradiation of the whole breast is a well assessed and effective therapeutic option in the treatment of localized breast cancer (1). Typically, more than 70% of women submitted to breast-conserving surgery receives post-operative radiotherapy and a large fraction of them is treated to sterilize the whole breast, typically delivering 38–40 Gy in 15–16 fractions or 50 Gy in 25 fractions. Due to the large and increasing incidence of breast cancer in the female population (2), this treatment represents a quite relevant fraction of the patients daily treated in the radiation oncology departments worldwide. Despite the evolution toward more personalized approaches including the possibility to reduce the treated volumes (i.e., partial breast irradiation) or to deliver higher dose to the tumor bed or to include selected nodal regions at risk, whole breast irradiation still maintains a central role in the treatment of breast cancer. Nowadays, different techniques are used; most Institutes still prefer the conventional tangential fields (TF) arrangement, either using 3-dimensional conformal radiation therapy (3DCRT) with wedges (physical or dynamic) or intensity modulated radiotherapy (IMRT), often delivered with a few segments per beam, mostly manually optimized (3–6). Rotational techniques (7, 8) generally showed better performances in better tailoring the dose distribution to the Planning Target Volume (PTV) shape with a consequent improved sparing of organs at risk (OARs) at high-intermediate dose levels, especially in the case of concave-shaped PTVs. Nevertheless, the issues related to the potential clinical meaning of the higher low-dose spread to the heart, lungs, and contralateral breast with rotational techniques is still open (9, 10). Due to this, the TF arrangement, limiting any relevant low-dose spread, is expected to stably remain among the most used techniques to treat breast cancer also in the next decade. On the other hand, manual (and also inverse planned) optimization is time consuming and highly dependent on the planner skill (11). Standardization in radiotherapy treatment planning is an important goal aimed to guarantee to all patients a high quality treatment, independent of the planner time and skills; this seems still more urgent in countries with a rapidly growing incidence of cancer and low/middle income (2). Automated planning solutions were recently introduced (12–25) with the aim of reducing planning time and inter-operator variability while conserving (or improving) a high quality plan (23, 26–29). Many systems were largely investigated for a different clinical situation including the breast site (11, 17, 30–35). Regarding the TF approach for whole breast irradiation, a relatively weak point of auto-planning is the difficulty to take into account the inter-patient variations in assessing the best position of the fields to limit the dose to the adjacent organs, concomitantly assuring PTV coverage and highly homogenous dose distribution within PTV. Consequently, automatic solutions for this application were rarely reported using in-house systems, intrinsically difficult to extend on a larger scale (11, 30, 32).

Automating the optimization of TF by knowledge-based (KB) approaches would be suitable as the (largely available) past information could be modelled to be optimally applied on new patients. The RapidPlan^®^ system (Varian Inc.) is available commercially and widely tested in many clinical scenarios, including breast VMAT (17, 31, 33). However, no clinical examples for the TF irradiation of whole breast are reported likely due to the configuration of the system implemented for IMRT/VMAT inverse-planning optimization. In order to obtain this objective, within a project for the large-scale implementation of automatic KB plan optimization (MIKAPOCo, Multi-Institutional Knowledge-based Approach to Plan Optimization for the Community), we previously demonstrated the possibility to mimic (and slightly improve) the performances of TF irradiation through a partially blocked multi-arcs approach using RapidArc^®^, named ViTAT [Virtual Tangential-fields Arc Therapy (36)]. Aims of the present work were:

1. to develop KB-models based on TF plans aimed to drive inverse-planned ViTAT plans; and
2. to demonstrate the possibility of replacing TF manual optimization with an automatic KB-ViTAT plan optimization.

## Material and Methods

### Clinical and Planning Procedures for Tangential Field Planning

At our institute, breast cancer patients treated with whole breast irradiation receives 40 Gy in 15 fractions (2.67 Gy/fr), prescribed as a mean dose to PTV. CTV and PTV are defined according to the AIRO national guidelines (36): in short, CTV is contoured excluding the skin, with a 5-mm margin from the surface while PTV is obtained by a 5-mm isotropic expansion. PTV is finally cropped with a 5-mm margin from the body surface. During the last 10 years, patients were treated mostly with opposed or quasi-opposed, BEV-based (Beam’s Eye View) optimized, fields, using physical wedges. Manually optimized segments were generally added to improve the PTV coverage and dose distribution homogeneity. Starting from 2018, this technique was gradually replaced by the manually optimized field-in-field technique (3), avoiding wedges. The number of segments for each field ranged between one (only open fields) and four, with most patients optimized using 2–3 segments per field. Moreover, for both techniques, the gantry position of few segments could be slightly changed (between 5° and 10°) with respect to the “prevalent” field, aiming to limit hot spots and improve homogeneity within PTV and OARs sparing. Based on the internally conducted plan comparison performed in 2017, the differences between FIF and our TF approach using physical wedges were clinically negligible, in slight favor of the FIF. Patients were treated with 6 MV beams, with few exceptions when 18 MV and 6 MV fields/segments were combined. During the last 5 years, all patients were treated with a Varian CLINAC-IX 2300 equipped with a 5-mm Millenium-MLC system; daily image-guidance with CBCT was performed for all patients; plans were optimized using the Varian Eclipse TPS system (v. 13.6) using the AcurosXB^®^ algorithm for dose calculation.

The planning goals for PTV were: V_95%_ > 95% (the fraction of PTV volume receiving more than 95% of the prescribed dose higher that 95%) and D_max_ < 108%. OARs were defined according to the AIRO national guidelines and always included the contralateral breast, the contralateral lung, the ipsilateral lung, and the heart: constraints were V_20Gy_ < 20% for the ipsilateral lung and V_5Gy_ < 40%, D_mean_ < 5 Gy for the heart in the left breast case. Independently from these constraints, planners always tried to reduce the dose to OARs as much as possible while respecting the goals for PTV. Contralateral breast was always avoided by the medial entry beam, while the lateral beam could include small portions at the exit. In the case the constraints could not be respected, PTV coverage received a higher priority against OARs sparing, with few exceptions. Alternatively, a VMAT plan could also be optimized and chosen by the physician.

### The ViTAT Technique

**Figure 1:**
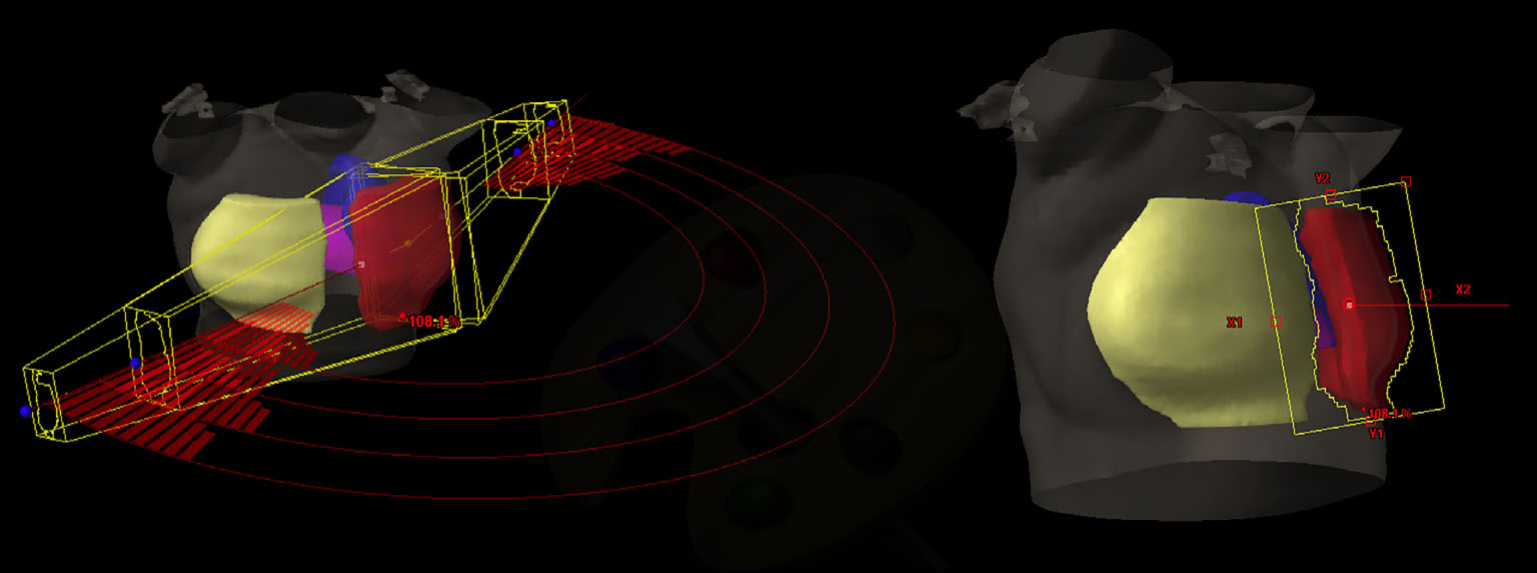
ViTAT setup: 3D view of a right side ViTAT plan showing the geometry of the four arcs used (on the left)—the segments show the beam delivery while the rest of the arcs are blocked for delivery; beam eye view of the medial angle (on the right).

As largely explained in Esposito et al. (36), we previously demonstrated the feasibility to mimic the TF irradiation performances through a multi-arcs approach where the delivery was partly blocked using RapidArc^®^ (RA), named Virtual Tangential-fields Arc Therapy (ViTAT). **Figure 1** shows the planning ViTAT set-up.

In short, ViTAT consisted of four arcs (6 MV) with a collimator rotation angle of ±10° and optimized with a RA technique with start/stop angles of 60°/220° for the right side and 300°/135° for the left one, consistent with the TF geometry in our population. Irradiation through the arcs was completely blocked, apart in the first and the last 20° of rotation. As for the breast VMAT-optimization (17), Target structure for plan optimization is obtained expanding the PTV outside the body (1.5 cm expansion in the external-lateral and anterior direction) to account for any breathing and inter-fraction deformation effect. A virtual bolus (1.5 cm thickness, -500 HU density) (17, 37) is linked to the fields during optimization to fill the Target structure during the optimization in order to avoid effects due to the electronic non-equilibrium and to assure a proper safety set-up margin (38, 39). Of course, the bolus is removed before the final dose calculation. Field dimensions are BEV-based adapted to the individual patient anatomy. Plans are optimized with the Rapid Arc (RA) optimization module by inverse planning. We implemented the ViTAT technique in this study with the goal of replacing the manual planning optimization in TF irradiation through a KB-optimization approach.

### KB-Model Generation

In order to guarantee a better plan homogeneity, TF clinical plans of the period 2016–2019 were considered for building the model, resulting in 90 plans (70 with wedged fields and 20 with FIF) for the right-side breast. In the same way, 103 (88 with wedged fields and 15 FIF) TF plans in the range 2016–2020 were selected for the left-side breast. These plans were used to train two KB-models, one for the right-side and one for the left-side breast using the RapidPlan (RP) tool implemented in the Eclipse TPS (v 13.6, Varian Inc.). This choice also balanced the need to train a sufficient number of plans and to keep a sufficiently large validation group, considering both the recent FIF (2019–2020) and older TF (before 2016) plans, as explained later. RP is configured to model inverse plans delivered with the IMRT/VMAT technique. Hence, each TF dose distribution was linked to a virtual RA-plan (40) consisting of two partially reverse arcs in the range 60°/220° for the right-side and 300°/135° for the left-side with a collimator rotation angle of ±10°. The choice of the start/stop angles was optimized according to the distribution of the beam angles of the original plans, as explained in (36). During KB modelling, arcs were not considered to be blocked (no avoidance sector were used), so the entire geometry of the structure is seen from the Beams Eye View (BEV) of the arc. Before accomplishing model configuration, the impact of using multiple arcs for building the models on DVH-estimates was investigated. As the partitions of the OAR during the training phase are the same for equal arcs, no differences were observed in the DVH-estimates for model configuration using two arcs instead of four ones (as used for the ViTAT techniques). The OARs considered for training the models were the ipsilateral lung and contralateral breast for the right side plus the heart for the left side.

Right-side heart and contralateral lung were not modelled as they were considered to be more easily managed in the final template (with the same fixed constraints for all patients) without any support of the prediction of the model, due to their anatomical position treatment and to the ballistic of the arc orientations. The tuning of the models was performed by using statistical tools available in the RP system and the Model Analytic platform: the methods followed to limit the impact of outliers were reported elsewhere (25, 41, 42). In short, for each OARs, the features that exceeded by >2 SD the principal components fitted with the KB-models were identified and the original clinical plan were individually re-evaluated; they were excluded as “dosimetric outliers” only in case of recognized sub-optimal planning. All the other potential outliers were found to be representative of an “uncommon” but clinically suitable geometry/anatomy condition and were kept in the models.

### Template for Automatic ViTAT Plan Optimization

Based on the resulting prediction models, the system generates the confidence intervals of the expected DVH for each OAR and suggests the lower confidence values to be used for plan optimization. In order to obtain a robust and efficient template, the choice of the position and penalty of the generated DVH constraints needs to be optimized. The optimization of the KB-based template for each side was carried out by several fine-tuning tests based on repetitive automatic plans for five sample patients for each of the two situations (right and left), testing the impact of the position of DVH constraints and their penalties, as similarly reported in previous studies (23–25). Based on our experience, once the PTV priorities are fixed, the OARs priorities were gradually increased through three KB-test templates. Selected relevant dose-volume parameters were analyzed for the five sample patients and compared against the original clinical plans to assess the performances of the three KB-based templates and to assess the one showing the best performances.

### Validation Tests

Validation tests were performed on 60 new patients equally divided between right-side and left-side breast. The external validation of the models was performed on patients selected in the range 2019–2020 and 2013–2016.In this way, we also intended to validate the model separately for wedged fields plans (the oldest group) and for the more recent FIF plans (delivered in 2019–2020). The number of FiF plans is 15 for right-sided breast patients and 11 for the left-sided ones, while the number of wedged fields is 15 for right-sided breast patients and 19 for left-sided ones. Importantly, the validation assessed the performances of the model to properly adapt the anatomical/morphological features of each individual patient and the feasibility of using TF DVH-prediction to fully automate the ViTAT optimization. According to others (17, 19, 23, 40), the validation was performed by re-optimizing a number of clinical plans and by comparing them against the original ones. All KB fully-automatic ViTAT-plans (KB-ViTAT) were compared against the original plans (TF) in terms of OARs/PTVs dose-volume parameters. The comparison was based on the analysis of the mean dose, maximum dose (D_1%_ for PTV and D_2%_ for OARs), and selected dose-volume parameters extracted from DVH. All selected parameters and DVHs were semi-automatically exported *via* ESAPI scripts and saved in spreadsheets for analysis. Wilcoxon-tests were performed to assess the statistically significant differences (p < 0.05).

## Results

In total, 10 patients (out of 90) for the right side and 18 patients (out of 103) for the left side were excluded by the model resulting in sub-optimal plans for at least one of the considered OARs or for PTV coverage. The final model returned χ^2^ and R^2^ parameters for the predicted OARs is shown in **Table 1**. The resulting DVH-estimates were used to generate an individually optimized KB-template for the ViTAT optimization. For the not trained OARs, the position of DVH constraints and their penalties were fixed and tuned as previously explained. Given the overall results, the KB-based template for automatic planning optimization was finally generated and is shown in **Table 2** for the right-side case and in **Table 3** for the left-side one.

**Table 1:** Final model goodness parameter for models for both right and left sided breast cancer patients.

Model	Structure	χ^2^	R^2^
**Right-side breast**	Ipsilateral Lung	1.043	0.604
	Contralateral Breast	1.050	0.511
**Left-side breast**	Ipsilateral Lung	1.043	0.723
	Heart	1.035	0.672
	Contralateral Breast	1.046	0.505

**Table 2:** The KB-based template for automatic planning optimization for the ViTAT technique for right-sided breast cancer treatment.

Organs	Objectives	Volume (%)	Dose (Gy)	Priority	gEUD a
**PTV**	Upper	0	40	500	
	Lower	100	40	500	
**Target**	Upper	0	40	500	
	Lower	100	40	500	
**Contralateral Lung**	Upper	0	3	600	
	Upper	2.5	1	150	
	Upper	10	0.7	150	
	gEUD		0.3	200	1
**Contralateral Breast**	Upper	Generated	1.5	600	
	Upper	Generated	1	200	
	Upper	0	Generated	400	
	gEUD		0.5	500	1
**Heart**	Upper	0	3	600	
	Upper	2.5	1	150	
	Upper	10	0.7	150	
	gEUD		0.5	200	1
**Ipsilateral Lung**	Upper	0	40	200	
	Upper	Generated	30	200	
	Upper	Generated	20	200	
	Upper	Generated	16	500	
	Upper	Generated	10	400	
	Upper	Generated	5	500	
	Upper	Generated	2	500	

The parameters obtained by the RapidPlan prediction automatically replace the “Generated” placeholder.

**Table 3:** The KB-based template for automatic planning optimization for the ViTAT technique for left-sided breast cancer treatment.

Organs	Objectives	Volume (%)	Dose (Gy)	Priority	gEUD a
**PTV**	Upper	0	40	600	
	Lower	100	40	600	
**Target**	Upper	0	40	600	
	Lower	100	40	600	
**Contralateral Lung**	Upper	0	3	600	
	Upper	2.5	1	150	
	Upper	10	0.7	150	
	gEUD		0.3	200	1
**Contralateral Breast**	Upper	Generated	1	500	
	Upper	Generated	1.5	250	
	Upper	0	Generated	450	
	gEUD		0.5	550	1
**Heart**	Upper	0	40	250	
	Upper	Generated	30	250	
	Upper	Generated	20	250	
	Upper	Generated	16	450	
	Upper	Generated	10	450	
	Upper	Generated	5	500	
	Upper	Generated	2	500	
	gEUD		3.4	500	1
**Ipsilateral Lung**	Upper	0	40	180	
	Upper	Generated	30	180	
	Upper	Generated	20	180	
	Upper	Generated	16	450	
	Upper	Generated	10	400	
	Upper	Generated	5	450	
	Upper	Generated	2	450	

The parameters obtained by the RapidPlan prediction automatically replace the “Generated” placeholder.

**Figure 2:**
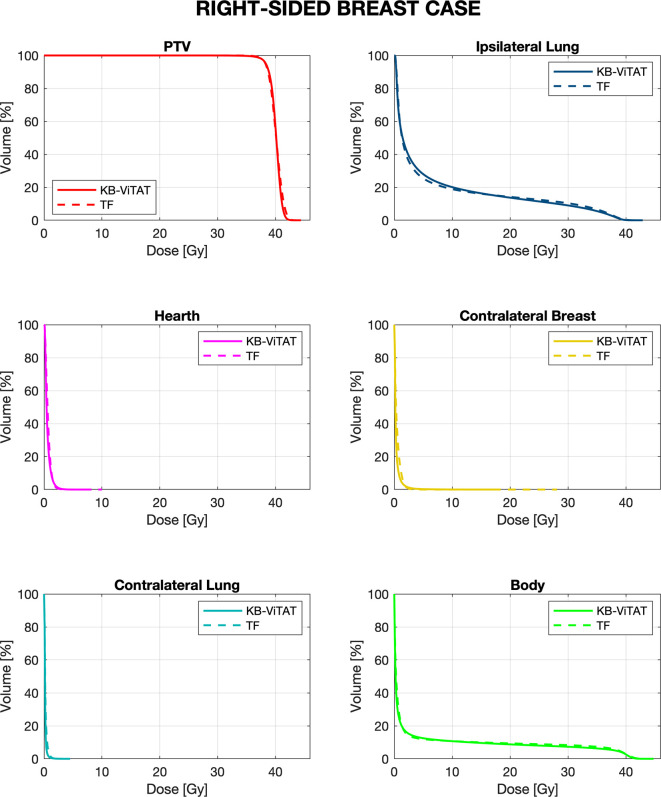
Mean-DVH comparison of 30 right-sided breast patient tests between the original clinical TF (solid lines) and fully automatic KB-ViTAT (dashed lines) plans.

**Figure 3:**
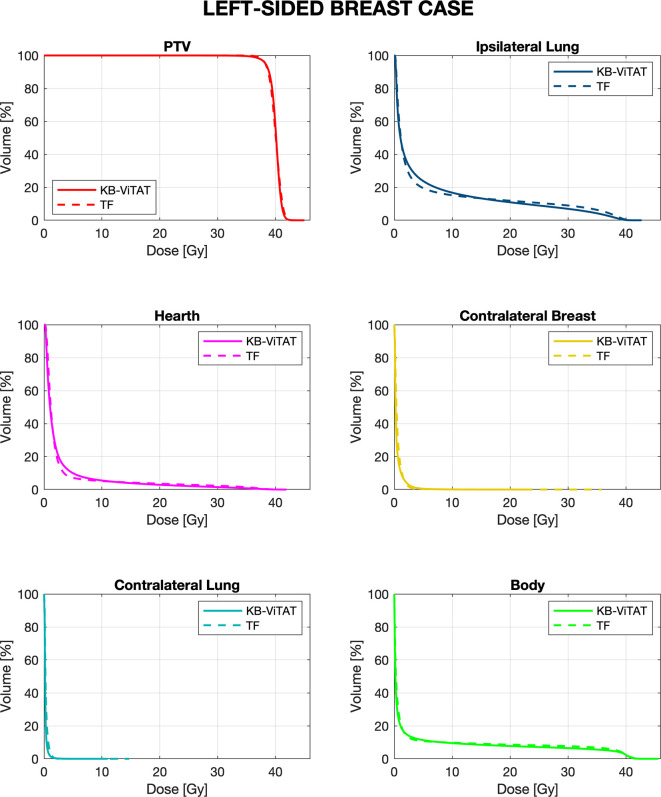
Mean-DVH comparison of 30 left-sided breast patient tests between the original clinical TF (solid lines) and fully automatic KB-ViTAT (dashed lines) plans.

Mean DVHs comparison between KB-ViTAT plans and clinical ones are shown in **Figures 2** and **3** for right and left side, respectively. On 30 KB automatic re-optimized cases per each side, only one for the right case and seven for the left case resulted in plans unacceptability in terms of the PTV coverage and/or ipsilateral lung constraints. The reason for this has to be found in the shape and position of the PTV with respect to OARs, causing an uncovering in the medial part of the PTV. However, after the manual refinement of the start/stop angles of 5°/10° by an expert planner, all automatic KB-ViTAT plans well fitted TF-performances. A manual refinement of 5° for the medial angle was necessary for the right-breast case and four of the left-side patients getting the following angles respectively: 65°/220° and 295°/135°. In the remaining three cases for left-side patients, it was necessary to also change the distal angle by 5° and 10° for two and one patients, respectively. Making the modification in the start/stop angles and re-starting the automated optimization, the PTV coverage and the ipsilateral lung constraints were satisfied and comparable to original clinical TF plan. As an example, **Supplementary Figures 1** and **2** (**Supplementary Material**) show the comparison of the originally automatic KB-ViTAT plan with the original start/stop angle and the refined KB-ViTAT with start angle modified by 5° (lower) for the right-side patient and for one of the left-side patients with manual modification of gantry angles.

Quantitative analyses are shown in **Tables 4** and **5**. Overall, differences between TF and KB-ViTAT were small and in slight favor of ViTAT. PTV coverage was similar, while PTV D_1%_ was improved with automatic optimization (p-value < 0.05). KB-ViTAT better spared contralateral OARs with respect to TF: mean dose was lowered by 0.1 Gy for all contralateral OARs, resulting in a decrease of 33% of the mean dose to the contralateral lung, 20% for the right and 20% for the left side contralateral breast, and 14% for the right-side heart.

**Table 4:** Dose-volume parameters comparison (TF vs ViTAT) for the validation cohort test of 30 plans for the right-sided breast case.

Organs	Features	*TF*	*KB-ViTAT*	*ΔP*
**PTV**	V_95%_ (%)	96.7 ± 1.3	96.7 ± 0.9	0.0
	D_1%_ (Gy)	42.3 ± 0.3	41.8 ± 0.3	**0.5**
	SD (Gy)	1.1 ± 0.1	1.0 ± 0.1	**0.1**
**Body**	D_mean_ (Gy)	4.2 ± 1.0	4.0 ± 1.0	**0.2**
	D_2%_ (Gy)	40.4 ± 0.4	40.2 ± 0.4	**0.2**
**Heart**	D_mean_ (Gy)	0.7 ± 0.2	0.6 ± 0.1	**0.1**
	D_2%_ (Gy)	1.8 ± 0.4	1.9 ± 0.6	-0.1
**Contralateral Lung**	D_mean_ (Gy)	0.3 ± 0.1	0.2 ± 0.1	**0.1**
	D_2%_ (Gy)	0.9 ± 0.4	0.7 ± 0.3	**0.2**
**Contralateral Breast**	D_mean_ (Gy)	0.5 ± 0.2	0.4 ± 0.2	**0.1**
	D_2%_ (Gy)	1.7 ± 0.6	1.9 ± 1.3	-0.2
**Ipsilateral Lung**	V_5Gy_ (%)	25.4 ± 4.8	28.0 ± 3.7	**-2.6**
	V_20Gy_ (%)	14.2 ± 2.7	13.7 ± 2.6	0.5
	D_mean_ (Gy)	6.8 ± 1.1	6.8 ± 1.0	0.0
	D_2%_ (Gy)	38.0 ± 1.1	37.8 ± 1.1	0.2

Parameters are presented as mean value ± standard deviation and differences ΔP. Values with a statistically significant difference (p-value < 0.05) are in bold.

**Table 5:** Dose-volume parameters comparison (TF vs ViTAT) for the validation cohort test of 30 plans for the left-sided breast case.

Organs	Features	*TF*	*KB-ViTAT*	*ΔP*
**PTV**	V_95%_ (%)	96.6 ± 1.5	96.3 ± 0.9	0.31
	D_1%_ (Gy)	41.9 ± 0.3	41.8 ± 0.3	**0.1**
	SD (Gy)	1.0 ± 0.3	1.0 ± 0.1	0.0
**Body**	D_mean_ (Gy)	3.9 ± 0.9	3.6 ± 0.8	**0.3**
	D_2%_ (Gy)	40.3 ± 0.3	40.1 ± 0.4	**0.2**
**Heart**	V_3Gy_ (%)	12.1 ± 6.1	16.5 ± 6.5	**-4.4**
	V_16Gy_ (%)	4.1 ± 2.1	3.6 ± 2.1	**0.5**
	D_mean_ (Gy)	2.7 ± 0.9	2.7 ± 0.9	0.0
	D_2%_ (Gy)	27.4 ± 9.9	23.3 ± 9.0	**4.1**
**Contralateral Lung**	D_mean_ (Gy)	0.3 ± 0.2	0.2 ± 0.1	**0.1**
	D_2%_ (Gy)	1.1 ± 0.4	1.0 ± 0.4	0.1
**Contralateral Breast**	D_mean_ (Gy)	0.6 ± 0.3	0.5 ± 0.2	**0.1**
	D_2%_ (Gy)	2.1 ± 1.2	2.8 ± 1.2	**-0.7**
**Ipsilateral Lung**	V_5Gy_ (%)	19.5 ± 5.9	23.8 ± 6.8	**-4.3**
	V_20Gy_ (%)	11.9 ± 3.9	10.9 ± 4.5	**1.0**
	D_mean_ (Gy)	5.7 ± 1.6	5.7 ± 1.7	0.0
	D_2%_ (Gy)	37.6 ± 3.1	35.9 ± 5.1	**1.7**

Parameters are presented as mean value ± standard deviation and differences ΔP. Values with a statistically significant difference (p-value < 0.05) are in bold.

The KB-ViTAT integral (i.e., body) dose was 5% lower than TF for the right case and 8% for the left case. Ipsilateral lung mean dose was identical (Right: 6.8 Gy, p > 0.05; Left: 5.7 Gy, p > 0.05): there was a modest worsening (i.e., few % volume) for the ipsilateral lung in the 2–15 Gy range and a slight improvement in the 20-35 Gy range when considering KB-ViTAT vs TF. The same behaviour was found for the left-sided case for the heart with a modest worsening in the range 2–10 Gy and a slight improvement in the range 15–35 Gy, while on average, delivering the same mean heart dose (2.7 Gy).

**Figure 4:**
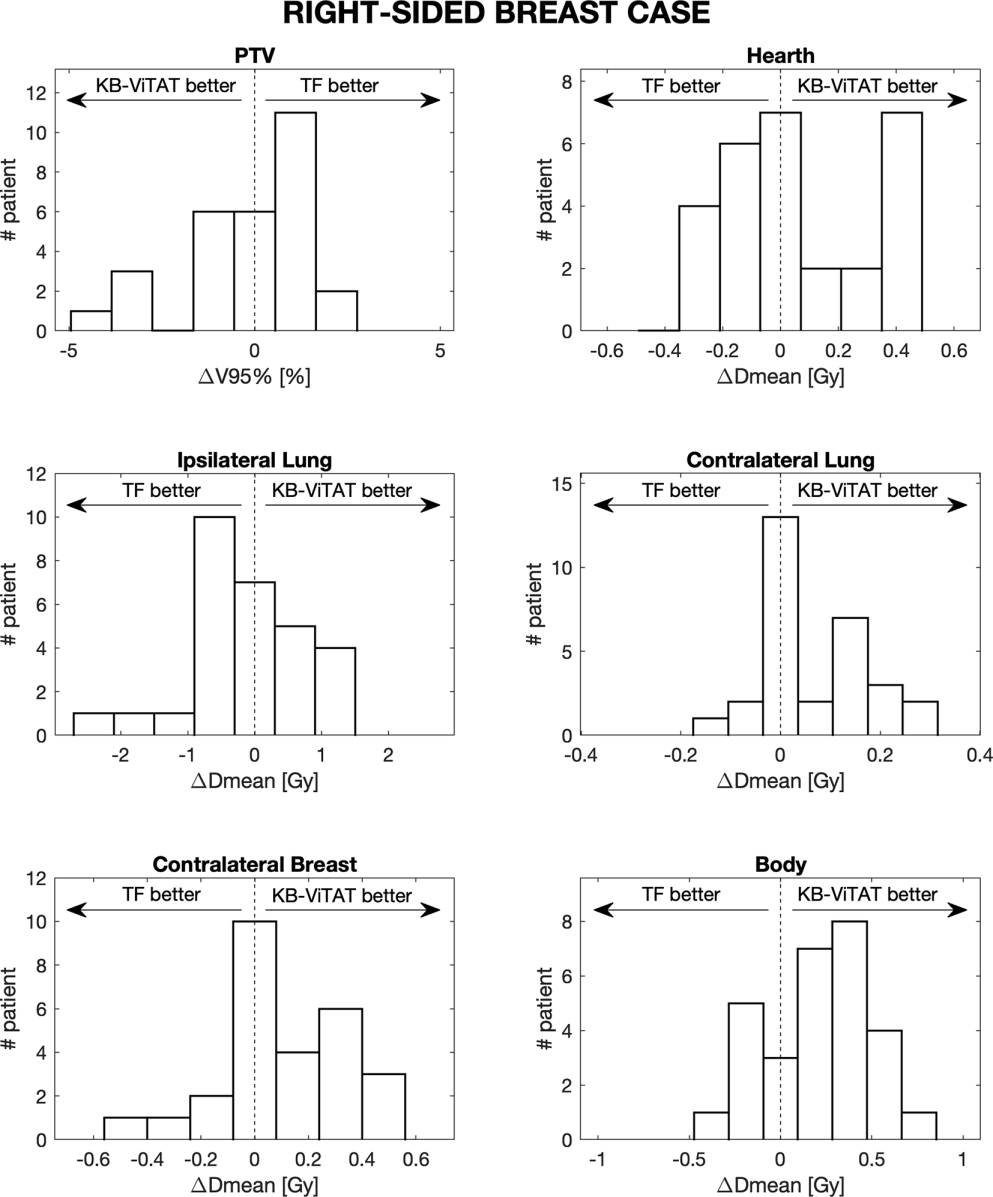
Population histograms of the differences between the clinical tangential field TF and automated re-optimized KB-ViTAT plans for the investigated dosimetric parameters for right-sided breast case, showing the V95% parameter for PTV and mean doses for OARs.

**Figure 5:**
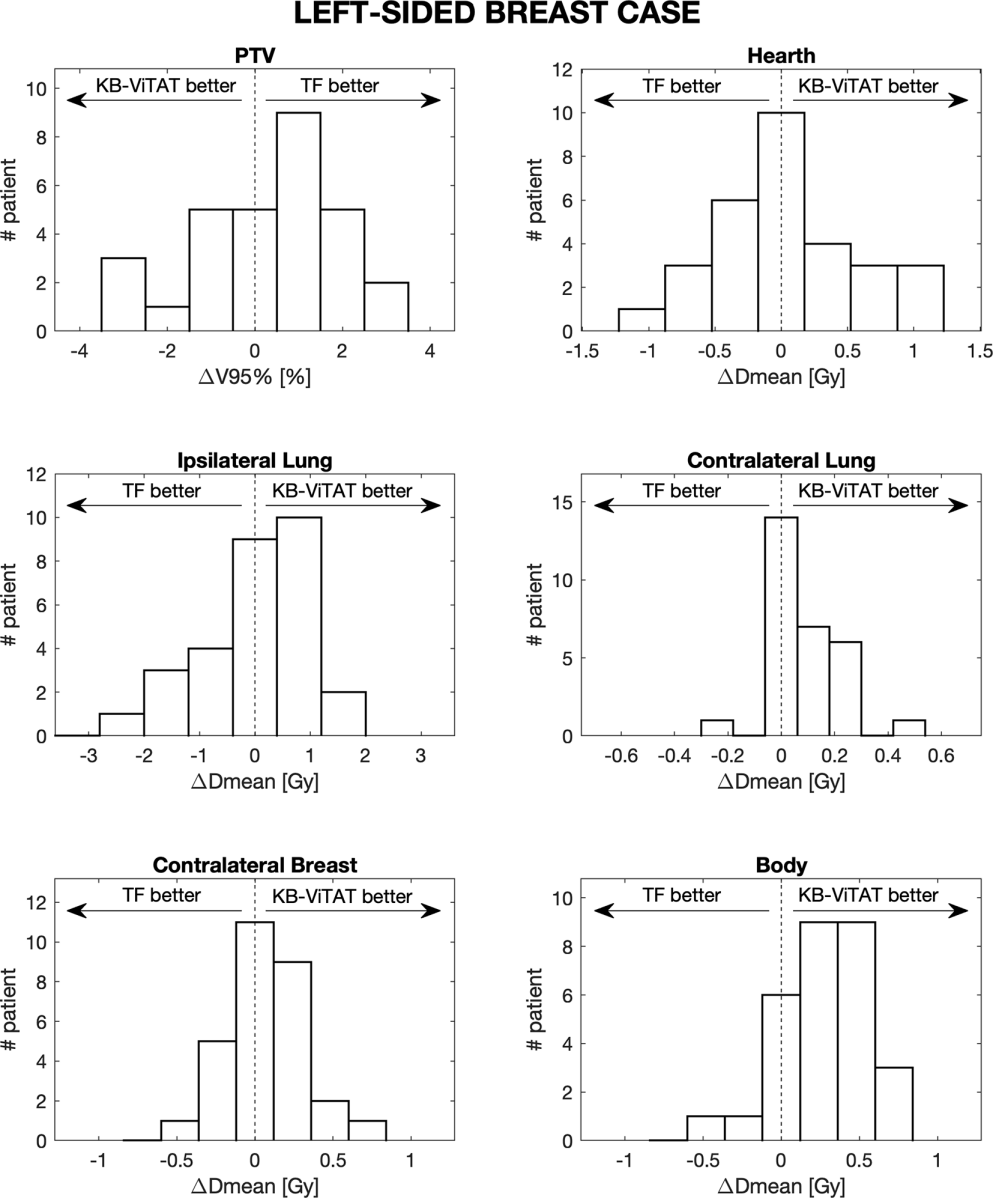
Population histograms of the differences between the clinical tangential field TF and automated re-optimized KB-ViTAT plans for the investigated dosimetric parameters for left-sided breast case, showing the V95% parameter for PTV and mean doses for OARs.

Furthermore, in **Figures 4** and **5**, the histograms representing the distribution of the differences between TF and KB-ViTAT were reported for the selected PTV/OARs dose-volume parameters. Overall, the time for automatic plan optimization and final dose calculation was registered and found to be 12 ± 2 minutes.

Importantly, the model performances were also evaluated separately for wedged fields plans (the oldest group) and the more recent FIF plans (delivered in 2019–2020). No statistically significant differences resulted in terms of the PTV/OARs dose-volume parameters when comparing the differences between KB-ViTAT and TF in the two cohorts of plans.

The KB-based automatic approach for ViTAT was clinically implemented, first for the right-sided breast and, more recently, for the left-sided one. Five plans for the right-side and five ones for left-side breast were preliminarily verified in terms of dose distribution in a planar phantom using a detector matrix. All clinical KB-VITAT plans underwent the same dosimetric verification before treatment. The gamma passing rate in comparing the calculated *vs* delivered dose maps was larger than 98% for all plans (in total n = 30), considering 3% - 3 mm as criteria, in agreement with our experience (43).

## Discussion

Scope of this work was to fully automate the planning of the whole breast treatment through the KB-optimization approach to mimic the performances of the tangential beam technique. The study aimed at assessing the capability of the RapidPlan tool to handle the dose distributions treated without using the VMAT technique. Moreover, we also explored the feasibility of translating the DVH-prediction model into a fully automatic optimized template for ViTAT planning. As previously shown (36), the ViTAT technique was able to generate dose distributions comparable to TF performances, with some slight improvement in the PTV coverage and homogeneity, in the sparing of contralateral OARs and with a mild reduction of the integral body dose. Then, as demonstrated here, once configured and validated, a KB model trained with TF dose distribution can be efficiently implemented for an automatic ViTAT optimization, completely and efficiently replacing manual plan optimization. The entire workflow was optimized aiming to automatize the selection of the beam angles through the VMAT approach but at the same time to avoid the dose-bath at intermediate–low doses typical of the rotational techniques (7). As a matter of fact, the concerns related to the increase of the mean dose to OARs dealt with the risk of increasing radiation-induced secondary malignancies and late cardiac events (1, 7, 44, 45). Few authors suggested a compromise between better conformity/OARs sparing at high doses against OARs sparing at intermediate–low doses to limit the dose bath associated to VMAT by the partial blocking of arcs (35, 46). Although all these approaches obtained a significant reduction of the low-dose bath, none of them followed the goal of mimicking tangential field irradiation, differently from our ViTAT approach. Other authors demonstrated the possibility of automatizing the angle selection for tangential fields (11) but none investigated the possibility of using the KB-approach. With the approach presented here, we demonstrated the feasibility of the complete replacement of manual TF plans with automatic plans with huge improvements in efficiency, in reducing/eliminating inter-planner variability, and in avoiding sub-optimal plans. Concerning this last point, despite a careful quantitative evaluation was not accomplished, it is relevant that about 15% of the clinical TF plans were *a priori* excluded when building the KB models as due to sub-optimal planning.

As a matter of fact, the automatic workflow that involved the fixed selection of the start/stop angles had proven to be an efficient way to reproduce the TF performances well. Only in about 13% of cases this approach had failed: on 60 KB automatic re-optimized cases, only eight resulted in plans that were unacceptable in terms of the PTV coverage and ipsilateral lung constraints. Seven out of eight referred to the left side were found to be more challenging from the point of view of the choice of the start/stop angles. However, with the manual refinement of the start/stop angles by an expert planner, the resulting automatic KB-ViTAT plans well fitted TF-performances also for these patients. Furthermore, plans were deliverable showing excellent dosimetric verification performances in phantoms. Importantly, a large sparing of planning time was obtained, with an overall time for automatic plan optimization and final dose calculation of 12 ± 2 minutes.

A highly relevant point, worthy to be underlined here, concerns the demonstration of the automation of a largely used technique by using a commercially available tool, making its potential adoption easy for Varian users. This issue is of primary importance, opening the possibility to a large-scale implementation with a consequent large reduction of the repetitive, manual procedures usually followed during a whole breast plan optimization. The possible sharing of KB-models, permitted by this system, should also be considered as an additional opportunity to rapidly spread this approach primarily to Varian users but in principle extendible/adaptable to other delivery systems (24, 40).

From a wider point of view, the large availability of TF plans and the “limited” inter-institute variability of PTV/OARs can make the development of robust KB DVH prediction models easier and, more importantly, the possibility of extending this approach on a multi-institutional scale. This issue is worthy of investigation and is currently under study within the MIKAPOCo (Multi-Institutional Knowledge-based approach to plan optimization for the community) consortium, joining several Italian Institutes.

Another relevant point concerns the possibility to exploit the ViTAT approach more to obtain further improved plan performances by the possibility of training models using the planning data obtained from clinical plans optimized and delivered at our Institute by applying more stressed intensity-modulation techniques: a project to train KB-models using the planning data of patients treated with Tomotherapy (using the Tomo-Direct module, resulting in IMRT-like TF plans) is currently ongoing.

## Conclusions

The approach followed here demonstrated the possibility of the complete replacement of manual tangential breast planning with automatic planning, including beam angle choice. Automatic fixed selection of the start/stop angles and KB-driven optimization were found to be an efficient way to well fit TF planning, with evident advantages in terms of time sparing, elimination of inter-planner variability, and of sub-optimal planning. Manual refinement of the start/stop angles was necessary in 13% of patients, with a large unbalance between the right (3%) and left (23%) sides, resulting in an additional 15–20 minutes more compared to the 12 ± 2 minutes spent for planning optimization and dose calculation in the remaining 87% of the patients. Due to its versatility and the use of a commercial system, this approach shows promising applications for a large-scale implementation.

## Data Availability Statement

The datasets presented in this article are not readily available because they are data of patients treated in a single center and cannot be shared without transfer agreement. Requests to access the datasets should be directed to fiorino.claudio@hsr.it.

## Ethics Statement

Ethical review and approval was not required for the study on human participants in accordance with the local legislation and institutional requirements. Written informed consent for participation was not required for this study in accordance with the national legislation and the institutional requirements.

## Author Contributions

RC, PE, and CF contributed with the conception of the study. RC, PE, and AT worked on data base and modeling. PM, SB, BL, and LP contributed in data integrity evaluation. CF, AF, SB, PM, NM, RC, and PE discussed the interpretation of the results. AF, CDA, NM, and MP were clinical responsible for the treatment of the patient. RC, PE, and CF wrote the manuscript. AV, NM, and CF reviewed and edited the manuscript. CF was responsible for the scientific coordination, managing, and project funding. All authors contributed to the article and approved the submitted version.

## Funding

The work was supported by an AIRC (Associazione Italiana Ricerca sul Cancro) grant (IG 23150).

## Conflict of Interest

The authors declare that the research was conducted in the absence of any commercial or financial relationships that could be construed as a potential conflict of interest.

## Publisher’s Note

All claims expressed in this article are solely those of the authors and do not necessarily represent those of their affiliated organizations, or those of the publisher, the editors and the reviewers. Any product that may be evaluated in this article, or claim that may be made by its manufacturer, is not guaranteed or endorsed by the publisher.

## Supplementary Material

Supplementary Figure 1 Comparison of plans between the originally automatic KB-ViTAT plan with start/stop angle of 60°/220° (upper) and the refined KB-ViTAT with start angle of 65° (lower) for the right-sided breast case. Starting angles are modified in order to obtain an acceptable coverage to PTV.

Supplementary Figure 2 Comparison of plans between the originally automatic KB-ViTAT with start/stop angle of 300°/135° (upper) and the refined KB-ViTAT with start angle of 295° (lower)for the left-sided breast case. Starting angles are modified in order to obtain an acceptable coverage to PTV and better hearth and lung dose distribution similarity between TF and KB-ViTAT case.
