# Supplementary figures and images for: Replacing Manual Planning of Whole Breast Irradiation With Knowledge-Based Automatic Optimization by Virtual Tangential-Fields Arc Therapy
Source: Front Oncol. 2021 Aug 24;11:712423. doi: 10.3389/fonc.2021.712423 (PMC8423088; doi:10.3389/fonc.2021.712423)

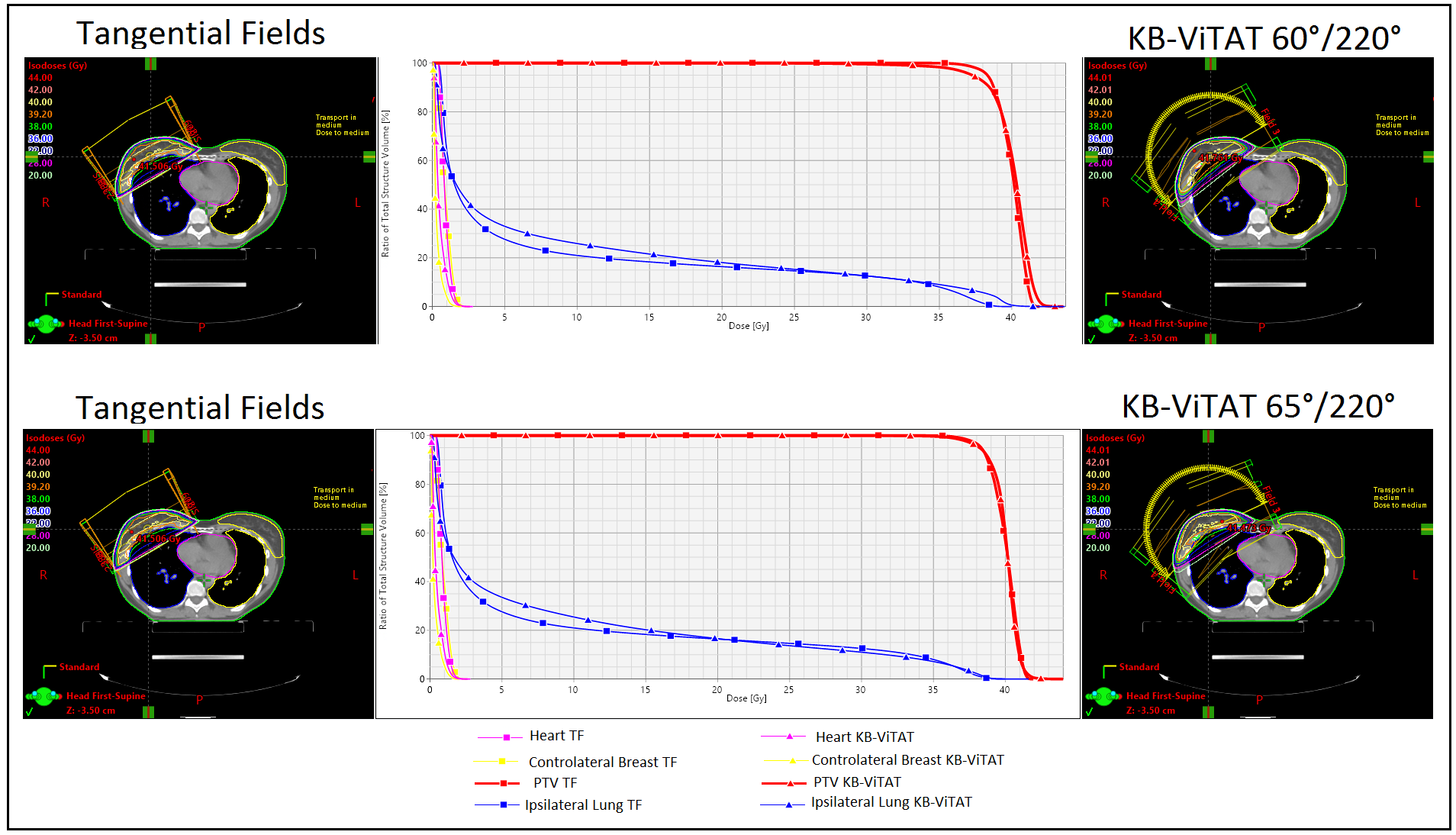

Supplement: Supplementary Figure 1 — Comparison of plans between the originally automatic KB-ViTAT plan with start/stop angle of 60°/220° (upper) and the refined KB-ViTAT with start angle of 65° (lower) for the right-sided breast case. Starting angles are modified in order to obtain an acceptable coverage to PTV. [file Image_1.tif]

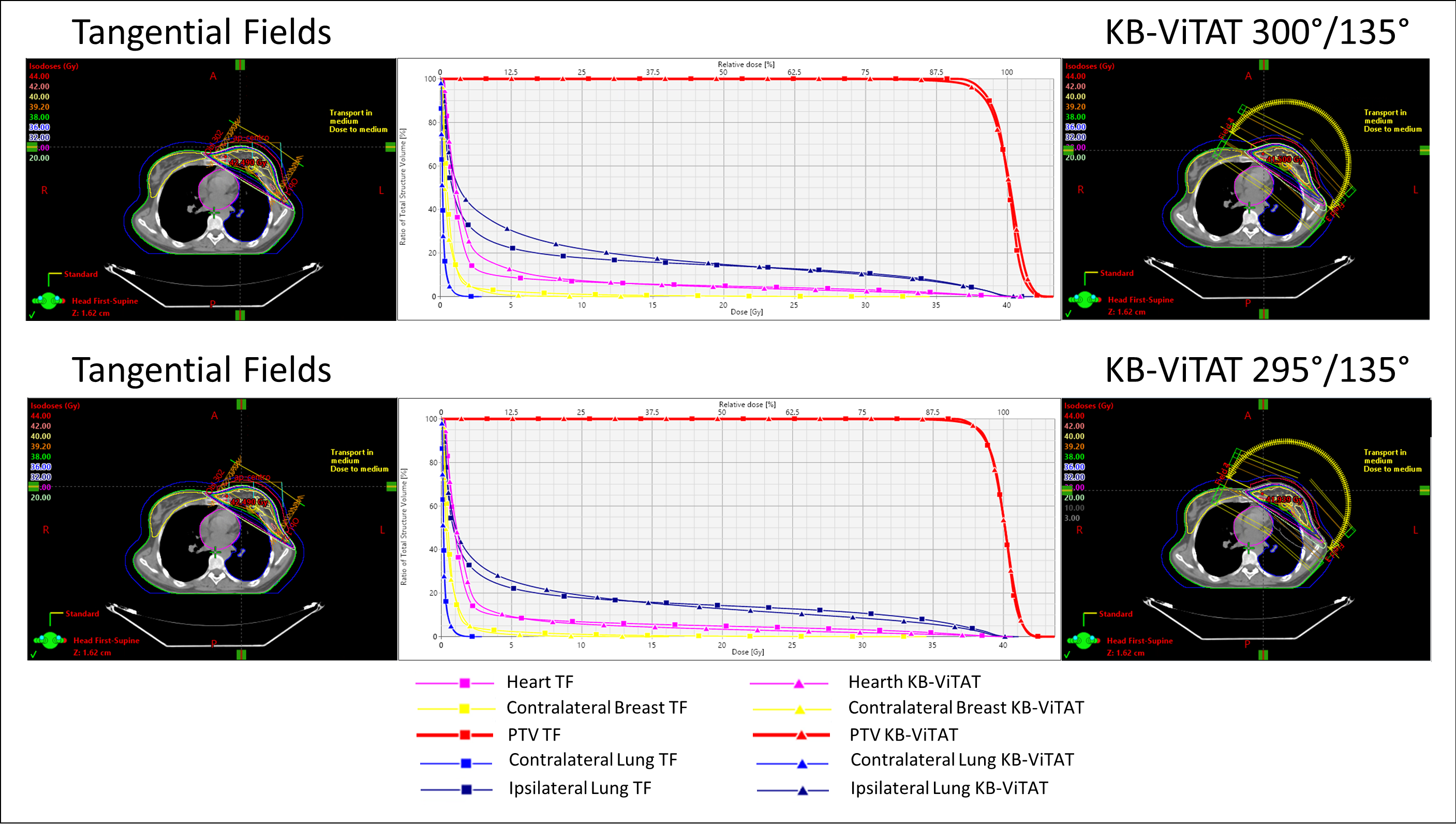

Supplement: Supplementary Figure 2 — Comparison of plans between the originally automatic KB-ViTAT with start/stop angle of 300°/135° (upper) and the refined KB-ViTAT with start angle of 295° (lower)for the left-sided breast case. Starting angles are modified in order to obtain an acceptable coverage to PTV and better hearth and lung dose distribution similarity between TF and KB-ViTAT case. [file Image_2.tif]
